# Analyzing the keys to the design of a mobile application for physical activity for school and out-of-school use from the perspective of adolescents, teachers, coaches, managers, and experts

**DOI:** 10.1371/journal.pone.0322074

**Published:** 2025-05-07

**Authors:** Adrián Mateo-Orcajada, Lucía Abenza-Cano, Pedro Ángel López-Miñarro, Lourdes Meroño, Ana María Gallardo-Guerrero, María de la Trinidad Morales-Belando, Noelia González-Gálvez, Alejandro Espeso-García, Tomás Abelleira-Lamela, Nerea Gómez-Cuesta, Antonio Joaquín García-Velez, Mario Albaladejo-Saura, Francisco Esparza-Ros, Raquel Vaquero-Cristóbal

**Affiliations:** 1 Facultad de Deporte, UCAM, Universidad Católica San Antonio de Murcia, Murcia, España; 2 Department of Didactics of Corporal Expression, Faculty of Education, University of Murcia, Murcia, Spain; 3 Kinanthropometry International Chair, Catholic University San Antonio of Murcia, Murcia, Spain; 4 Department of Physical Activity and Sport Sciences, Research Group Movement Sciences and Sport (MS&SPORT), Faculty of Sport Sciences, University of Murcia, San Javier, Spain; Ahvaz Jundishapur University: Ahvaz Jondishapour University of Medical Sciences, IRAN, ISLAMIC REPUBLIC OF

## Abstract

No previous research has analyzed the opinion of adolescents, teachers, coaches, managers, and mobile app experts, on the usefulness and functionality of mobile apps for use by adolescents. For this reason, the objectives of this research were: to discover their opinion about the physical activity apps currently available; and to determine the elements and characteristics that they consider most relevant to find in a physical activity app that can be used for a longer period of time. Eight focus groups were carried out in which a total of 38 adolescents (mean age: 13.74 ± 1.24 years old), 29 teachers, coaches, and managers (mean age: 35.27 ± 2.81 years old), and 10 experts (mean age: 43.18 ± 4.22 years old) participated. The most relevant results regarding the apps available include limitations in their functionality and design, as well as in the information provided and the requirements, which means that they are not designed exclusively for adolescents. Regarding the demands for a specific app for adolescents, the participants highlighted gamification as the main element, as it is key to user engagement, as well as the possibility of recording physical activity along with other healthy habits. Challenges, competitions or the possibility of observing progress should also be present in the application as they also influence user engagement and motivation. Adolescents also highlighted a multimedia section, privacy and rewards, while for professionals the inclusion of feedback, the facilitation of work and a fast interface for use in the school environment were key.

## Introduction

The use of mobile apps to promote the practice of physical activity in the adolescent population has gained relevance in recent years [1,2]. This is because these tools have been shown to be effective in increasing the level of physical activity in this population [3,4], as well as in obtaining physical and body composition benefits [5–7].

Despite the effectiveness shown by mobile physical activity apps [8](Lee et al., 2019), previous research has shown that the adherence of adolescents to this type of intervention is low [9,10], with the use of these devices decreasing considerably after the first few weeks, when the initial novelty wears off [11]. In addition, another of the main problems encountered with the use of mobile apps is that those currently on the market, although used and shown to be effective in adult populations [12], are not designed for the adolescent population [13].

These handicaps hinder the independent use of apps by this population in their free time [6]. Previous studies have shown that a complex interface, not knowing how to use the application, a lack of interest, and the fact that the application does not work on the mobile devices of adolescents, given the high technical specifications required by most of them, are some of the reasons why almost 50% of adolescents abandon their use within a few weeks of starting [10]. On their use in the school context, previous research has shown that these apps have a complex interface that limits the possibilities of use in the educational classroom [14]. Thus, the mobile apps available have failed to capture the attention of adolescents [10], and do not make it easier for teachers to make an appropriate use of them in the classroom [14].

Previous research has analyzed what aspects must be included in a mobile physical activity app to increase its use by adolescents [15], but it has only considered its use outside school hours, leaving aside the possibility of turning it into an educational resource with a place in the school environment. Moreover, previous studies have neither considered the opinion of professionals working with adolescents (teachers, coaches, sports managers), nor experts in the development of mobile apps, who could offer an important perspective in terms of interface and functionality from that provided by adolescents. For this reason, the aims of the present research were a) to discover the opinion of adolescents on the mobile physical activity apps currently available on the market for physical activity practice; b) to discover the opinion of teachers, coaches, managers and experts in the field, on the mobile apps currently available on the market for use with adolescents; c) to determine the elements and characteristics that adolescents consider necessary to include in a physical activity mobile app so that its use is constant over time; and d) to establish the elements and characteristics that teachers, coaches, managers, and experts, consider necessary in a physical activity mobile app for adolescents to facilitate its implementation.

This study aims to answer the following questions: What do adolescents, teachers, coaches, managers, and experts think about the physical activity mobile apps currently available on the market? And what aspects do adolescents, teachers, coaches, managers, and experts consider necessary to include in a physical activity mobile app so that it is designed for adolescents and its use is constant over time?

## Materials and methods

### Design and participants

This was a qualitative study using a focus group approach in which students, teachers, coaches, managers, and experts were recruited. All focus groups with students were carried out with adolescents from an accessible compulsory secondary school. In order to carry out the study, the school’s management team was contacted and, after obtaining their approval, the relevant information was disseminated among the students by means of an information sheet sent via the virtual campus and posted on the school bulletin board. A face-to-face meeting was held with all the students interesting in participate, following the methodology of previous research [16,17]. After that, a list of those teenagers who wanted to participate in the study was generated and students completed the Physical Activity Questionnaire for Adolescents (PAQ-A) [18], in its Spanish version [19] to determine the level of physical activity of the adolescents before the focus groups. This was followed by the application of the inclusion criteria among participants. The inclusion criteria for the students were as follows: a) being enrolled in compulsory secondary education; b) aged between 12 and 16 years old; and c) having previously used physical activity mobile apps.

As a result of the above, there was a total of 604 student volunteers to participate. After that, adolescents were classified according to gender and level of physical activity performed with these data. Once classification was performed, a random selection of students for the focus group was made based on the heterogeneity of profiles, following the methodology from previous research [16], and giving rise to four focus groups, thus ensuring an adequate representation of males and females, and of active and inactive adolescents: a) physically active males and females; b) physically inactive males and females; c) physically active and physically inactive males; and d) physically active and physically inactive females. So, a total of 38 students participated in the four focus groups (18 males, 10 active and 8 inactive; 20 females, 9 active and 11 inactive), with ages between 12 and 16 years old (mean age: 13.74±1.24 years old), following the sample sizes of similar research in this field [20,21].

About the focus groups with teachers, coaches, managers, and experts, contact with them was made through social and mass-media channels, and by email, in accordance with the methodology of previous research [17]. Thus, announcements were placed on the social networks of the participating institutions and/or research groups, and emails were sent to databases of the networks and universities participating in this study. In the case of teachers, coaches, and managers, the inclusion criteria were as follows: a) working in the field of sport or education with adolescents aged between 12 and 16; b) not having participated in previous focus groups on the subject; and c) having previously used physical activity mobile apps. In the case of experts, with the understanding of an expert as a person who has information and knowledge in a substantive area beyond that of the average person, and who regularly shared this information and knowledge through consultation, teaching, or public speaking, or publications and written reports [16], the inclusion criteria were as follows: having previously taught, researched, or developed mobile physical activity applications. In both cases, participation was not limited according to the age of the professionals as they could bring totally different visions of the educational use of technology as they were from different generations.

After this procedure, there was a pool of 189 teachers, coaches and managers; and 39 experts who were classified according to gender. The selection of people for the focus group was made according to the heterogeneity of profiles, following the methodology of previous research [16], and avoiding gender biased [22]. So, men and women were randomly selected, ensuring homogeneous participation in terms of gender to be part of four focus groups: a) male teachers, coaches, and managers; b) female teachers, coaches, and managers; c) mixed teachers, coaches, and managers; and d) mixed mobile app experts. A total of 29 teachers, coaches, and managers participated in the focus groups (14 males and 15 females; 11 teachers, 10 coaches, and 8 managers; mean age: 35.27±2.81 years old). In addition, 10 experts participated in the research (mean age: 43.18±4.22 years old). These sample sizes are similar to those of previous research in which focus groups were conducted with this population [23,24].

The recruitment period for the study was from 15 October to 10 November 2023 for teachers, coaches, managers, and experts, and from 3 May to 20 May 2024 for adolescents. Participants were informed of the procedure and aim of the study, and any questions they might have about the study were answered. Prior to the start of the study, the participants provided a signed informed consent to participate in the study and to use the data obtained anonymously for scientific purposes only. In the case of participants who were minors, the informed consent was also signed by their parents. Participants were also informed that they could leave the research at any time during the study and that their data collected thus far would be deleted. The study design and protocol were approved prior to initiation by the institutional ethics committee of the Catholic University of Murcia (code: CE022102), following the principles of the World Medical Association and the Declaration of Helsinki. The design also followed the STROBE guidelines [25].

### Focus groups and discussion topics

The research design was consistent with a critical realist epistemology, which allowed for adequate knowledge and understanding of what is happening in the field of mobile applications for physical activity through the experiences of adolescents, teachers, coaches, managers and experts [26,27].

The focus groups were conducted in Spanish and were composed of between 6 and 10 participants, which is an appropriate sample size to ensure adequate information collection from the group dynamics and interaction between participants. This number of participants and the heterogeneity present in each focus group increases the representativeness and validity of the results [16]. A soundproof room was used for the focus groups, with the participants seated in a circle, favoring interaction between participants and an adequate hearing of all contributions. All sessions were conducted by a single researcher, who was impartial and neutral with respect to the opinions given, avoiding taking a position that would influence opinions [28–30]. Two researchers collaborated in recording the sessions with a password-encrypted digital recording device to facilitate subsequent analysis. The duration of each focus group was standardized between 60 and 90 minutes, thus avoiding that the time factor could interfere with the results obtained [30].

Prior to the start of the focus groups and following the methodology of previous research [17], a group of 10 experts with previous experience in the design and use of this methodology designed the questions that would guide the discussions of the adolescents and the teachers, coaches, managers, and experts. For this purpose, a three-hour face-to-face meeting was held to discuss the questions to be included in both focus groups. This meeting resulted in a draft of questions that were discussed again in a second meeting two weeks later with the same group of experts. The meeting resulted in the final questions for the focus groups (Table 1).

**Table 1:** Questions asked by focus group moderators.

Students focus group
1. Have you ever used mobile apps for physical activity? For what purpose (personal use, physical education class, training log)? Alone or accompanied by family, friends, etc.?
2. What did you like most about the physical activity mobile apps you have used? And what did you like least?
3. Of the mobile apps used, do you think they were designed for you or people your age? Why? And do you think they are designed more for males, females or both equally?
4. Do you think mobile apps can help you to be healthier? Why?
5. Would you like to be able to use a mobile physical activity app to record what you move both in and out of school? Why?
6. What aspect of physical activity would you log at school and outside school?
7. What would you like to see in a mobile app so that you would use it more often?
8. What aspects related to healthy behaviors would you like to find in a mobile app?
9. Would you like the physical activity mobile app to be a game where you have to do physical activity to achieve rewards or goals? What would you include in that game?
10. Do you share or would you share the training done with the mobile app on social media? Do you allow, or would you allow this information to be public or would you prefer to keep it private?
**Teachers, coaches and managers focus group**
1. What is your previous experience with the use of mobile apps for physical activity in the subject of physical education? And outside of it?
2. Which mobile apps in particular have you used in physical education classes? Why did you choose these apps over other similar ones?
3. Do you consider current mobile apps useful for improving students’ adherence to healthy lifestyle habits? Why?
4. What positive aspects would you highlight about the use of mobile apps for recording physical activity in the school setting? And outside the school setting?
5. What do you think are the limitations to the use of mobile apps in the physical education class? And the limitations to their use outside the school setting?
6. Do you think there are differences in the use of apps depending on gender? Who uses them more (males or females)? Why? Do you think the apps are designed with one gender more in mind than the other?
7. Do you think that there are differences in terms of adherence between mobile apps with and without gamification? Why?
8. Do you think that the mobile apps currently available on the market are adapted to the needs and interests of adolescents? Why?
9. What information do you think would be relevant to include in a mobile app to promote a healthy lifestyle among adolescents?
10. What is needed for physical education teachers to integrate mobile physical activity apps in physical education classes?
**Experts’ focus group**
1. Have you ever thought about, researched, or developed mobile physical activity apps? What are their main advantages and disadvantages?
2. What aspects should be improved in the physical activity apps currently available on the market if they are to be used by adolescents?
3. What do you consider important to improve adherence to the apps?
4. Do you think that the reward systems within the apps favor their use and generate adherence on the part of adolescents? What type of rewards?
5. Do you think that the apps currently available are more oriented to one gender than to another? Why? In case of an affirmative answer, what should be done to make these apps usable by anyone?
6. Do you consider that the inclusion of healthy habits could be relevant to increase adolescents’ adherence to these apps? Why?
7. What elements would you consider essential to encourage the use of this type of apps by adolescents? And by teachers?

The questions followed a semi-structured approach, as they included leading questions, flexible probing questions, and clarification questions, as had been done in previous research [31]. The structure of the focus groups allowed the researchers to discuss different areas of perceived importance [32].

The focus groups encouraged collective discussions. The goal was to build upon and challenge ideas, ultimately reaching a consensus on a series of questions specifically developed by a group of experts, following established methodologies [16]. According to previous research, the participants’ subjective viewpoints and experiences were recorded during the focus groups, as well as their intentions, hopes, concerns, feelings, and beliefs. In addition, the participants’ opinion was about the external topic, not including demographic queries about income or other personal information [16].

At the beginning of the focus group sessions, the researchers made a presentation about the research and the associated project, according to the methodology of previous research [16]. After this, the participants made a short introduction in order to get to know each other and facilitate interaction during the focus group. Once the presentation was finished, the questions designed for each focus group were asked in the established order. Once the question was posed, the participants were free to participate and interact, sharing their experiences and personal anecdotes, questioning each other, supporting, or disagreeing with the opinions given, but without making value judgements on any participant, in accordance with the focus group’s operating procedures. No participant was obliged to take part in the questions, nor was there a pre-established order of intervention, so participation was free and voluntary. However, the moderator ensured that participation was as balanced as possible among the participants, according to previous research [16,17].

To ensure the truthfulness and authenticity of the data collected during the focus groups, an environment of trust was fostered in which participants felt comfortable sharing their perspectives in an honest manner. In addition, data triangulation was applied, comparing and contrasting the information obtained between the different focus groups to identify consistent patterns and possible discrepancies [33]. Researcher triangulation was also implemented, involving multiple analysts in the data interpretation process [34], which allowed us to reduce potential individual biases and enrich the understanding of the findings from diverse professional perspectives.

The implementation of eight focus groups also made it possible to obtain a more complete perspective of the phenomenon due to the heterogeneity of the sample, as well as to discover the opinion of the participants, avoiding the influence of aspects such as gender or the practice of physical activity. The observance of the study’s fundamental ethical principles of benefit, fairness, and conscience, and the anonymity of study participants with encrypted information was ensured. All of the above aspects contributed to the attainment of valid and reliable data on the subject in question, in line with previous research [28,29].

### Data analysis

The design, analysis and presentation of the results followed the recommendations of previous studies based on qualitative methodology and more specifically on focus groups [35–37]. The analysis design established by Braun and Clarke [38] was followed to systematize the codification, identification themes and interpretation these themes in relation to the research questions. The thematic analysis conducted was an inductive thematic analysis [39,40], as every piece of text was coded. Open coding was used, with no pre-established codes, developing and modifying the codes as the coding process progressed. Two researchers independently performed a deep analysis of the data, first reading through all the focus group transcripts. They conducted multiple coding sweeps, initially focusing on surface meaning and later on latent meaning. Each researcher independently generated themes from the established codes. After completing this work separately, they collaborated to revise and refine the codes and themes, critically examining each other’s assumptions and perspectives [28]. A third reviewer was consulted to resolve any disagreements. Finally, the researchers coded all the focus group translations according to the established codes and themes. Themes were actively developed through patterns of shared meaning, and were inductively derived, rather than being developed based on the topic guide. Anonymized quotes were extracted from the data to support the themes. The NVivo 12 Pro software was used to develop codes and themes during the reflexive thematic analysis [40].

## Results

### Thematic analysis

**Fig 1:**
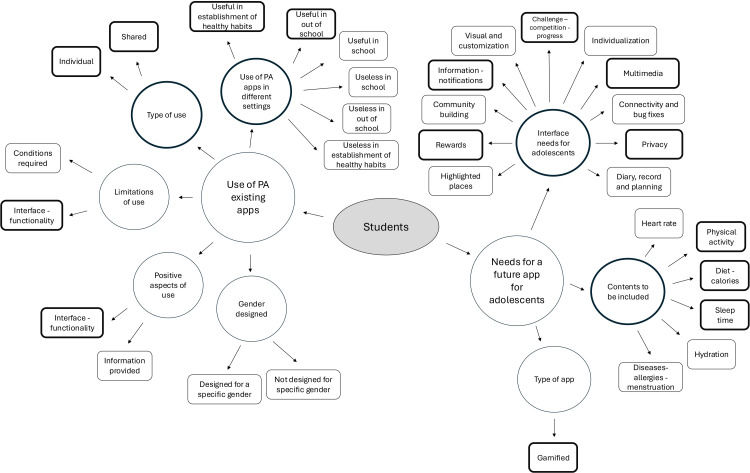
Graphic representation of NVivo coding in the student focus groups.

**Fig 2:**
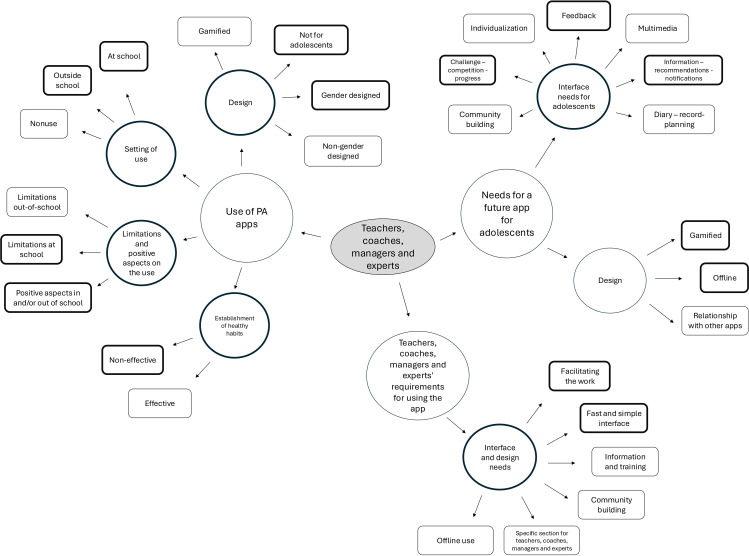
Graphic representation of NVivo coding in the teachers, coaches, managers, and experts focus groups.

Participants’ ideas and reactions were assigned codes. Where necessary, subcategories of a theme were created to indicate that it was nested within a broader idea or concept. The results on the constructs of the focus group of students and the focus group of teachers, coaches, managers and experts were presented separately. All quotes were contextualized through the use of codes. Figs 1 and 2 show the coding process, showing the areas with overlap and the most cited areas (bold). These themes, groups and codes were established after the following process: 1) initial reading of text; 2) identify specific text segments related to objectives; 3) label the segments of text to create categories; 4) reduce overlap and redundancy among the categories; and 5) incorporating the most important categories. All this was always carried out by two investigators who always sought concordance.

### Student’s analysis

The codes of the student focus groups were established. From these codes, two themes were obtained. The first referred to the opinions and experiences of the students when using physical activity apps that were already available on the market (“use of physical activity existing apps”) (n=181 citations), with five groups into it: use of physical activity apps in different settings (n=97 citations); type of use (n=32 citations); limitations of use (n=18 citations); positive aspects of use (n=16 citations); and gender designed (n=18 citations) (Fig 1 and Table 2).

**Table 2:** Themes, groups, and codes identified in the students focus groups.

Theme	Group	Code	Males	Females	Total	Cite
Use of PA existing apps	Type of use	Individual	9	10	19	‘Personal use’. ‘I use it alone for sport’.
		Shared	5	8	13	‘With family, friends or in PE classes’. ‘There is a mountain routes app that I use every weekend with company’.
	Limitations of use	Interface and functionality	5	6	11	‘They are quite boring and monotonous’. ‘It has to be more specific, because if you don’t go running or do any sport, it doesn’t count your steps’. ‘Some of them are very bad and don’t record the distance travelled well’.
		Conditions required by the app	3	4	7	‘You have to enter your email or your personal details’. ‘You have to give your location’. ‘You have to pay for it’. ‘It uses up a lot of battery and takes a lot of storage/space’.
	Positive aspects in the use of apps to record PA	Interface and functionality	4	5	9	‘When you don’t feel like going out, you can do sport at home’. ‘They are usually quite intuitive exercises to do indoors and outdoors’. ‘It allows you to use it in physical education’.
		Information provided by the app	3	4	7	‘They provide a lot of information: stress, sleep, physical activity’. ‘They include videos of how to do things. It gives you instructions’. ‘It makes routines adapted to your age’. ‘It keeps a record of what you have done each day and the route in case you want to repeat it’.
	Use of PA apps in different settings	Useful in out of school settings	11	13	24	‘To record what I do in a sport’. ‘To know what I’m walking, to have a control’. ‘To know throughout the week how much you have done’.
		Useful in the establishment of healthy habits	11	13	24	‘The app motivates you to keep exercising and not give up. It sends you a reminder’. ‘Yes, it helps us to be healthier’. ‘Many times you don’t know how to do something, and the app helps you to do it well’.
		Useful in school setting	8	7	15	‘Yes, because in high school we also do physical activity in PE, at break time’. ‘In PE we all do the same thing and there is no difference, but you can record break time because there are competitions between classes’. ‘If you set a goal to do x steps a day, you can also count the steps at school because we also move around here’.
		Useless in school settings	8	6	14	‘The signal is lost very quickly, and they are not useful. ‘Inside the school it’s not useful, outside it is’. ‘At school you can’t use the mobile’.
		Useless in out of school settings	5	6	11	‘I don’t find it interesting to use an app to record physical activity outside school’. ‘Between scooters, buses and cars, no one walks to school or to school’.
		Useless in the establishment of healthy habits	5	4	9	‘It depends on the application you use; it can be a good option or not’. ‘It depends on whether the person creating the application knows about it or not’.
	Gender designed	Designed for a specific gender	3	8	11	‘There are some apps that are designed more for males and females. For example, the flexibility apps are more oriented towards females and the icon shows females’. ‘There are apps where the exercises are only for females, such as legs or waist. Also, the figures in the app show men or women depending on who they are aimed at’.
		Not designed for a specific gender	3	4	7	‘They are designed for both equally’. ‘The step counting apps are designed for everyone’.
Needs for a future app designed for adolescents	Contents to be included	Physical activity	9	7	16	‘There are many sports that can be recorded, cycling, paddle tennis, etc.’. ‘You can differentiate between exercises inside and outside the classroom’.
		Diet/calories	7	7	14	‘It allows you to record your diet. Calories, what you eat’. ‘That it gives you information, not only about calories, but also about the macronutrients in your diet’. ‘Food can be scanned, and you can know what you are eating’.
		Sleep time	6	7	13	‘To record the hours of sleep’. ‘It tells you how much time you spend in each sleep phase’. ‘The nap. That allows you to set special alarms to comply with a healthy nap’.
		Heart rate	5	4	9	‘To record heart rate during the day’. ‘If you are exercising and your heart rate goes too high or too low, let me know’. ‘To create a record of several days and, based on the average of those records, to make recommendations for you’.
		Hydration	3	4	7	‘Allowing me to record my hydration level’. ‘Tell me how much water I need to drink’.
		Diseases/ allergies/ menstruation	0	5	5	‘Ask about pain, mood’. ‘To consider whether menstruation is regular or irregular’. ‘That it has a section to enter if you have illnesses, allergies, etc.’.
	Type of app	Gamified	10	5	15	‘Flexibility, reflexes, or agility could be included through games’. ‘For people of our age it could include games so that we don’t get bored using it’.
	Interface needs for adolescents	Challenge/ Competition/ Progress	21	16	37	‘It should include competitions within the school, but also regional, national, and even international competitions’. ‘There could be categories according to the level of each user and match users based on this’. ‘There could be a weekly challenge and everyone in the app could participate, and a ranking could appear’. ‘Depending on your progress, it can level you up. That it detects your level and adapts’.
		Multimedia (Social network, photos, videos)	10	14	24	‘Like a social network, like TikTok, Instagram, but for sport. Allow you to share routes or places you’ve visited’. ‘It should allow you to upload photos’. ‘That you can share what is related to PA. But no other types of content like on other social networks’. ‘That you can share the improvements achieved by using the app. When you achieve it, a button to share it directly appears’.
		Privacy	13	11	24	‘That it allows you to adjust when information is recorded and when it is not’. ‘That you choose whether you want to share what you have done with others or not. Allow you to choose your privacy’. ‘That the settings for sharing are very clear and simple. They are always hidden, and it makes it difficult’.
		Information/ notifications	9	11	20	‘To tell you if what you have run is right or wrong according to your level’. ‘That it includes reminders to do exercise’. ‘That, if I look for routines to do, you get different alternatives from the app and from other people with comments on whether it’s good or not’. ‘That the app gives valid information, and you don’t have to rely on advice from friends or family’.
		Rewards	10	8	18	‘Set a goal and when you reach it, it gives you a reward’. ‘Get paid for walking. They give you points, and you can exchange them for things in the app’. ‘That there are locked options within the app and that they unlock as you do more activity (emojis, stickers, animals)’.
		Community building	6	7	13	‘Do it with friends. You can invite friends to do a specific challenge to exercise together’. ‘That it allows you to make friends within the app’. ‘That bad comments and insults are automatically blocked’.
		Visual and customization	7	6	13	‘Include a lot of visual content’. ‘Avatars and emojis should be very visual’. ‘That it allows you to create an avatar. Instead of a picture of yourself, you put an avatar’.
		Individualization	5	7	12	‘That you can choose what you want to improve. To tell you the consequences of choosing one program or another’. ‘That you can enter your age, weight or height and based on this it makes recommendations for food and PA’. ‘That it is in several languages for people from abroad’.
		Connectivity and bug fixes	6	5	11	‘That the settings are easy to see and manage’. ‘Let me select whether I want to receive news or not’. ‘That it can be used without Wi-Fi or internet. That it saves data until you connect to the internet again’. ‘That it can be connected to a watch or fitness wristband’. ‘That it responds to comments, complaints and suggestions made. Customer service’.
		Highlighted places	3	2	5	‘To show you routes in the mountains, because if you download the app, it’s because you like sport and you want to know where to do sport’. ‘It can recommend areas where you can do routes’. ‘That you can mark the places where you go hiking, trekking, the routes you do’.
		Diary/ Record/ Planning	3	2	5	‘That in a section of the app it includes what you have done today, at home or outside, and what you have to do tomorrow’. ‘That it allows you to write down the amount of weight you move in the gym’.

Within the group “type of use”, the codes “individual” (n=19 citations) and “shared” (n=13 citations) were established. In the group “limitations of use”, the code “interface and functionality” (n=11 citations) was the most important. In the group “positive aspects of use”, the codes “interface and functionality” (n=9 citations) was highlighted. Within the group “use of PA apps in different settings”, the codes “useful in out of school settings” (n=24 citations) and “useful in the establishment of healthy habits” (n=24 citations) were the most cited (Fig 1 and Table 2).

The second theme included the opinions and suggestions that this population would like to find in a physical activity app that was designed specifically for them (“needs for a future app for adolescents”) (n=261 citations), with three groups: interface needs for adolescents (n=182 citations); contents to be included (n=64 citations); and type of app (n=15 citations) (Fig 1 and Table 2).

In the group “contents to be included”, the codes “physical activity” (n=16 citations), “diet/calories” (n=14 citations) and “sleep time” (n=13 citations) were the most cited. In the group “interface needs for adolescents”, the codes “challenge/competition/progress” (n=25 citations), “multimedia (social network, photos, videos)” (n=24 citations), “privacy” (n=24 citations), “information/notifications” (n=20 citations) and “rewards” (n=18 citations) were highlighted (Fig 1 and Table 2). Finally, in the group “type of app”, the code “gamified” (n=15 citations) was found.

### Teachers, coaches, managers, and experts’ analysis

In the case of teachers, coaches, managers, and experts, the codes gave rise to three main themes. The first theme was “use of physical activity apps” (n=168 citations), with four groups: setting of use (n=40 citations); limitations and positive aspects on the use (n=47 citations); design (n=55 citations); and establishment of healthy habits (n=26 citations) (Fig 2 and Table 3).

**Table 3:** Themes, groups, and codes identified in the teachers, coaches, managers, and experts focus groups.

Theme	Group	Code	Males	Females	Total	Cite
Use of PA apps	Setting of use	Outside school	11	8	19	‘Hiking routes can be made and a final montage with the videos and images that have been created’. ‘The inverted classroom can be used so that pupils can work on the content at home’. ‘A club can be created for each class in the school, competing with each other after school hours’.
		At school	8	5	13	‘To create PA challenges to practice during class’. ‘You can use an app that is similar to Pokémon Go, with the teacher putting cards with QR codes that the students have to scan to get the benefits’.
		Nonuse	3	5	8	‘It is difficult because they do not have the necessary devices’. ‘Apps are not integrated into teaching units’. ‘The use of apps and bracelets is quite low in PE classes’.
	Limitations and positive aspects on the use	Limitations at school	9	10	19	‘Many schools prohibit the use of mobile devices, so a lot of data is lost’. ‘It is not known if they actually use the app or if they go to other apps’. ‘If we introduce apps every day in every physical education class, we are moving away from the real aim of getting them active’.
		Positive aspects in and/or out of school	8	8	16	‘It can improve strength, flexibility and general physical abilities’. ‘It can be a good way to attract children to physical activity’. ‘The app can be used in the classroom to teach them how to record steps’. ‘They can try to achieve the physical activity guidelines they are taught at school through the app’.
		Limitations out-of-school	6	6	12	‘It is difficult to control their use of apps. Some of them modify the parameters so that they count steps without moving’. ‘In some sports, they can’t wear apps or wristbands. Therefore, the time of day when they do the most strenuous activity cannot be recorded’. ‘Many parents limit the use of mobile phones for their children, and sometimes students do not have a mobile phone and have to ask their parents for one’. ‘The protection of student data is fundamental. Many apps include GPS that tell you where you start walking and where you end’.
	Design	Not for adolescents	8	11	19	‘They do not include gamified or attractive environments for use by adolescents’. ‘The apps are for adults and our motivation is not that of an adolescent. We need to change the perspective and make them for adolescents’. ‘They adapt to interests, but not to needs. They take them away from real life and social skills’.
		Gender designed	7	8	15	‘The purchase of fitness wristbands for PE class was proposed, and the female students refused because they were not aesthetic’. ‘Fitness apps are more targeted and more used by males, while food apps are more used by females’. ‘In adolescence, there are differences between males and females in the practice of physical activity. It is not so much the device, although females are more inclined to wear fitness wristbands’.
		Non-gender designed	6	6	12	‘Apps are for everyone. It’s not because you are a female or a male that you are offered one thing or another. It depends on what the person is looking for’. ‘The differences in usage are minimal between males and females, but because they don’t use them a lot’.
		Gamified	4	5	9	‘Gamified attracts more attention. Challenge and reward’. ‘Gamification includes a story that increases motivation’. ‘If it is gamified and played in community, all the better’.
	Establishment of healthy habits	Non-effective	8	9	17	‘Some of the exercises or recommendations that appear in the apps are not healthy’. ‘They can become obsessed with achieving aims, achievements, with being the best, and this can lead adolescents to problems’. ‘Adherence and dependence go hand in hand. Be careful about how much time they spend using apps’. ‘In some apps reality is distorted, they show something that is not real, and this can have a negative effect on young people’. ‘These apps provide them with models that are not appropriate and make them adopt unhealthy lifestyle habits’.
		Effective	5	4	9	‘They are useful, but we have to see if they are safe or not in the educational sphere’. ‘I think it is not negative that these apps are based on extrinsic motivation or achievements. It is true that students can get frustrated if they are always at the bottom of the ranking, but we must also teach them to manage this’. ‘Based on the adherence of this population to the mobile phone, if we get them to spend time on the phone but invest it in healthy behavior, we will achieve benefits’.
Needs for a future app designed for adolescents	Interface needs for adolescents	Information/recommendations/notifications	9	6	15	‘That it includes information about nearby sports centers, places to practice physical activity.’. ‘Notifying you about the benefits. What happens to my body by doing this physical exercise’. ‘That it includes news based on scientific evidence. That it educates adolescents with quality information’. ‘That it includes the technical movements of certain exercises that capture the students‘attention’. ‘Information, but also about the negative aspects, about what if you do it, it takes away from you’.
		Feedback	6	7	13	‘Instant feedback on what is being done’. ‘Giving feedback on what they have done is rewarding. We give them this kind of information and motivate them’. ‘Feedback. Providing information about the consequences of the activity’.
		Challenge/Competition/Progress	7	5	12	‘It should include challenges that award points that can be exchanged in real life for small prizes’. ‘It must have a reward, so that they can see that what they have done has served a purpose’. ‘It’s not just about competition, it’s about seeing progress. If they do more physical activity, they get more points’. ‘Compete against yourself and also against others would be key.’ ‘You can also compare with other peers, with teachers. A reference that motivates them to do the activity’.
		Community building	4	5	9	‘It is important to include families, because the environment is key’. ‘Some people like to see what their peers have done, so creating a community can be a good option’. ‘Create communities, something that engages and allows them to stay connected. There should be an adult managing the community’. ‘There should be a group with the teacher where doubts can be solved’.
		Multimedia (Social network, photos, videos)	3	2	5	‘Photos, likes, notifications, attractive interface, all of this motivates usage in this population’. ‘Allowing them to upload photos or videos of what they have done’.
		Individualization	2	3	5	‘You have to look at the initial level, it is not the same if they are active or inactive at baseline. You have to individualize’. ‘If you design apps with individual short-term aims or that include physical activity bracelets, you can introduce the habit of walking’.
		Diary/Record/Planning	3	2	5	‘Anything that quantifies PA is a motivator, both in quantity and intensity’. ‘I would include how you have felt every day when you do sport. The perception’. ‘They don’t have to enter data every day. The data should be entered automatically’.
	Design	Offline	5	6	11	‘It would be important to be able to work offline, to upload the data and then transmit it to the app when they have a connection’.
		Gamified	3	4	7	‘Gamification could be used as an evaluation system. Depending on the badges earned with the app’. ‘Gamified challenges could be done with the whole class. Everyone can contribute what they can, but they all add up’.
		Relationship with other apps	3	2	5	‘A mechanism could be included in the app so that, when you start doing PA, it does not let you use any other app on your mobile’. ‘Connecting the PA app with other ‘social’ apps and allowing you to share your achievements in the PA app with the other apps’.
Teachers, coaches, managers and experts’ requirements for using the app	Interface and design needs	Facilitating the work	7	7	14	‘It should make our work easier, not be an additional burden. If you have to spend two or three hours a day managing it, you’re not going to use it’. ‘It should help teachers with programming. They should be able to integrate it easily’. ‘It (should) generate a database automatically in which the data of the students associated with a specific class is recorded’.
		Fast and simple interface	4	7	11	‘We need a quick, easy, accessible and simple interface for us and for them. Currently they are not easy to use, they are not intuitive’. ‘We need it to be fluid and not stop all the time. You can’t assign prizes, tasks or rewards if the app keeps crashing’.
		Information and training	3	4	7	‘We need to be trained, because nowadays these devices are very integrated in our daily lives, and we need to be updated’. ‘We need to be trained to know how apps work so that we can integrate them into our daily classroom life’.
		Community building	3	4	7	‘Include contact between different schools through the app. This makes it possible to generate joint challenges between schools’.
		Specific section for teachers, coaches, managers and experts	3	3	6	‘A specific section for PE teachers in which to include ideas for classes and which allows everyone to obtain knowledge and new ideas for classes’. ‘The app should include a section with all the content and the teacher should be able to choose which content to work on each day’. ‘It should be organized by course. That very specific content can be included for each course’.
		Offline use	2	3	5	‘It would be very interesting to be able to work offline. It would be very interesting if data could be uploaded and then transferred when we are connected’. ‘We don’t need mobile all the time. It should be possible to use it offline’.

The “design” group was composed for “not for adolescents” (n=19 citations) and “gender designed” (n=15 citations) as highlights. Within the group “setting of use”, the codes “outside school” (n=19 citations) and “at school” (n=13 citations) were highlighted. In the group “limitations and positive aspects on the use”, the codes “limitations at school” (n=19 citations) and “positive aspects in and/or out of school” (n=16 citations) were the most cited. In the group “establishment of healthy habits”, the codes “non-effective” (n=17 citations) were highlighted.

The second theme was “needs for a future app for adolescents” (n=87 citations) with two groups: “interface needs for adolescents” (n=64 citations) and “design” (n=23 citations) (Fig 2 and Table 3).

The group “interface needs for adolescents” is composed of the codes “information/recommendations/notifications” (n=15 citations), “feedback” (n=13 citations) and “challenge/competition/progress” (n=12 citations), among others. Within the “design” group, the codes “offline” (n=11 citations) and “gamified” (n=7 citations) were highlighted (Fig 2 and Table 3).

The third theme was “teachers, coaches, managers and experts’ requirements for using the app” (n=50 citations), with a unique group: “interface and design needs” (n=50 citations). Into this group can be found the codes “facilitating the work” (n=14 citations), and “fast and simple interface” (n=11 citations) as key groups (Fig 2 and Table 3).

## Discussion

The first and second objectives of the present study were to discover the opinion of adolescents and teachers, coaches, managers, and experts on the mobile physical activity applications that are currently available for the practice of physical activity. About the use of physical activity apps in different settings, it is worth noting that both students and teachers, coaches, managers, and experts referred to the usefulness of these devices in out-of-school hours. Previous research has shown that mobile apps become more useful and relevant when used in the out-of-school setting due to their greater applicability [13]. This is probably due to the fact that these devices are not prepared to be used at school [14], with some of the problems identified in previous research being difficulties in synchronizing the devices [41]; and teachers are unable to integrate them into physical education teaching units [42]. This finding is similar to previous research in which the extracurricular use of these physical activity apps is beginning to be promoted from physical education subject [4]. In addition, the positive aspects of using these apps in out-of-school hours to record physical activity could be the improvement in students’ physical capacity, which is consistent with previous research [4]; the apps allow them to establish a physical activity routine [43]; the objectivity and immediacy of the data [44,45]; it allows students to include their family or friends in the achievement of their goals with the app [46,47]; and it serves as a complement to the teaching and learning process of students [48].

One point of disagreement between students and teachers, coaches, managers and experts was whether these apps could be effective in improving adolescent health. In this respect, students considered that existing apps on the market could be useful to establish healthy habits. Previous research has shown that when these applications include general advice on physical activity [49,50], motivate people to be physically active [45], or include information about other healthy habits [51,52], showing to be beneficial for increased physical activity [53]. The results found in the present research agree with previous research, as the inclusion of healthy habits in the app was considered an effective strategy by the vast majority of participants. However, it should be noted that professionals consider that existing app are not effective for establishing healthy habits in adolescents. This could be due to the fact that the apps have major limitations in terms of the credibility of the health recommendations included, as they lack professional supervision and credible sources, which diminishes their credibility [54–56]. Another limitation in this field is that previous research has shown that the use of lifestyle apps may be related to increased problematic use of these devices [57]. In addition, one of the main problems of these apps is that they do not allow to control the intensity [58], which is a fundamental variable to obtain improvements in health [59], so their usefulness seems limited. This view of professionals about the lack of benefits that adolescents could obtain from the use of this type of apps may be another reason why their use and promotion is reduced [60].

It should also be noted that there was no consensus among both the students, and teachers, coaches, managers, and experts on the issue of apps designed for a specific gender. They indicate that that fitness apps are more targeted to men, while those that include social networks are designed for women. Previous research showed that the usage patterns of males and females may be different within the app [61]. In this line, males tend to use apps for improving muscle mass to a greater extent than females [62] and this could lead to focus group participants having this view that apps are gender biased. In addition, it is interesting that in the step-counting apps, no differences were found, indicating that they are designed for everyone. Aerobic exercise is more universal and has similar aims in terms of improving cardiorespiratory fitness, so there are fewer gender differences. Given the interesting results of this research, this is an area that still needs to be explored in future research.

Regarding the target group, there were no doubts among teachers, coaches, managers, and experts as the vast majority considered that they were not designed for adolescents, mainly highlighting interface issues, the lack of gamification, and the inclusion of motivational aspects for adults, but not for adolescents. These results are in line with previous research, which indicated that these apps are not designed specifically for adolescents [14], and that their future design should consider the target group, as meeting personal expectations would increase usage [63].

Not in vain, students indicated that there were limitations in terms of interface and functionality in the current app available on the market. These results are in line with previous research that show that one of the main reasons for not using the mobile apps was their complex interface [10]. This previous research also showed that functionality was one of the categories to which adolescents gave the lowest scores [10], and the fact that the app did not work on the mobile device [64,65], or that it uses a lot of battery were negative conditioning factors [45,66].

Regarding the type of use, it should be noted that there were disparate opinions among adolescents, since a considerable number of adolescents used it alone, while others used it with their family or friends. Previous research has also shown that adolescents use these apps alone and accompanied [5,67]. The fact of using them alone is due to the fact that they acquire a greater perception of autonomy to select and monitor their physical activity [5]; while when using them accompanied, it has been observed that the increase in physical activity is greater by involving close members and increasing the motivation to practice [68]. So future research needs to focus on this issue.

In response to the first research question regarding what adolescents, teachers, coaches, managers, and experts think about the mobile physical activity apps currently available, in terms of design, apps in general are not specifically designed for adolescents and it can be stated that adolescents observed limitations in the functionality and design of the apps. With regard to the setting of use, both adolescents and teachers, coaches, managers, and experts clearly indicated their potential for use outside school. Finally, there are discrepancies in the potential of the apps to generate healthy habits, where teachers, coaches, managers, and experts indicate that and not effective in establishing healthy habits; while adolescents think that it could be a resource to achieve this.

With regard to the third and fourth aim, to determine the elements and characteristics that adolescents and teachers, coaches, managers, and experts, consider necessary to include in a physical activity mobile app for adolescents so that its use is constant over time and to facilitate its implementation in different fields, the results show that both students and teachers, coaches, managers, and experts indicated that it is not only relevant to include the recording of physical activity, but that they would also like to find other healthy habits such as eating and sleeping. Previous research has shown that the acquisition of one healthy habit could favor the acquisition of others [69], and it could be the case that including physical activity in the app could lead to the acquisition of other healthy habits, or vice versa. This is of great relevance, as it would allow adolescents to achieve a state of comprehensive health, complementing the practice of physical activity with other healthy habits [70]. In addition, this could lead adolescents to use the app to a greater extent, as it would not be reduced only to recording the physical activity practiced, granting a greater versatility to the app, which is consistent with previous research in which function combinations, rather than standalone functions, contributed to app success [71].

In addition to the above, it should be noted that teachers, coaches, managers, and experts indicated that it is essential to include the ability to monitor progress and feedback on the activities carried out, through which users receive immediate information about the physical activity they have just practices. This is in line with the opinion of adolescents reported in previous studies, in that personalized feedback on performance was an important aspect of continued use of physical activity apps and other apps related to healthy habits [2,72,73]. One possible explanation for this is that feedback provided by teachers, coaches and peers has a positive effect on engagement [74,75], so the same could occur with the feedback provided by the app. This finding further highlights the importance of including feedback in such mobile apps, as it seems to have a very positive impact on motivation and engagement. So much so that apps that include feedback mechanisms increase the level of physical activity to a greater extent [76]; increase well-being [77]; and maintain engagement for longer [78]. Some of the mechanisms that can be introduced in these apps and that have been shown to be effective in previous research are personalized feedback, understood as personal suggestions based on the recorded data [79]; social feedback, in which comparisons of steps with peers are made, which can motivate the user [80]; or goal setting with feedback on progress, which shows the user his or her progress in the set challenges [81]. Therefore, the inclusion of elements that provide feedback in the app is a very good option because it does not imply a high increase in the app’s requirements and the benefits obtained are high in the psychological field, mainly in terms of motivation and engagement.

Regarding to the type of app, both adolescents and professionals referred to gamification as a key aspect for the app. The importance of gamification lies in several aspects: the relationship between gamification and the motivation, competence, or autonomy of adolescents [82,83], as well as on adherence and engagement [84,85], facilitating the acquisition of a routine [86,87]; the autonomy granted through the ability to customize and create one’s own character [15]; or the existence of a virtual world that encourages the practice of physical activity [88,89]. In fact, previous research using gamified mobile apps has shown benefits on body composition, physical fitness and psychological state of adolescents [89–91], which reinforces the importance of gamified mobile apps. Some elements that could be included would be points and rewards; competition; the possibility to customize and personalize; or the visual appearance [92,93]. All these aspects can have a considerable impact on adolescents’ playability and engagement, however, one of the main limitations of these apps is that they do not work on all devices due to the requirements they need [64,65]. Therefore, including these elements could be interesting, but it should be really assessed whether this possibility would increase usage or make it even more difficult.

Also in this interface needs topic, both adolescents and professionals showed great interest in finding challenges, competitions and rewards. These results are similar to those of previous studies showing the important role that competition and rewards plays for this adolescent population. In this regard, competition can drive engagement, allowing comparison with peers, which can motivate them to perform above the rest [94]. In addition, rewards are a powerful motivator, as they provide immediate gratification and recognition for the achievement of the goals set, which makes adolescents respond positively to them [5]. And, with respect to challenges, these provide a sense of accomplishment and progression, which is key to maintaining interest and motivation, especially in apps that offer various levels of difficulty and new challenges [95]. The combination of these three elements generates a dynamic and interactive environment that can keep teens interested in this type of apps [15].

It is interesting that adolescents see the need for a multimedia section where they can find photos, videos and ways to interact with others, as well as create communities within the app. This is similar to previous research, where was observed that one of the main preferences of adolescents in mHealth apps is that they offer peer support through social media [93]. A possible explanation for this could be that social media allow adolescents to develop emotionally, reducing the perception of loneliness and increasing the ability to relate to others [96], as well as to shape their social identity and improve their overall well-being [97,98]. It is also worth noting that in education, social media brings benefits related to early learning, exposure to new ideas and increased opportunities for social support and contact [99], which makes this issue even more relevant. However, there is another crucial aspect to consider that is closely related to multimedia: the privacy of these applications. Although adolescents claim to be able to show their achievements, share images or videos, and determine whether this content is public or private, this is an issue that generates controversy, also in previous literature [100,101], so the inclusion of this type of content in physical activity apps should be evaluated in depth.

Furthermore, a relevant aspect indicated by professionals is the possibility of using the app offline, and that the data can be loaded later when there is an internet connection. The importance of this lies in the fact that, for teachers, the app would truly become an educational tool that would serve as a complement to physical education classes, as sometimes the full potential of these apps is lost in the absence of a stable internet connection [102]; while for adolescents, the offline app would allow them to maintain a level of physical activity without the need to access the internet, making it easier to integrate exercise into their daily routine, regardless of connectivity [5]. For this reason, future app design needs to prioritize this over the creation of a community building or multimedia space, as apps are already available.

In terms of the needs that should be considered in the app for teachers, coaches, managers and experts to use it with adolescents, a fast and easy interface, and facilitation and automation of the teacher’s work stood out. What teachers seek when they include these tools in the classroom is a facilitator of their work, not an impediment that reduces students’ active time. In this sense, it seems more efficient to use a simple app, with a fast interface and the minimum necessary functionalities, than an app with an excess of functionalities that is slower [15]. Furthermore, it is important to highlight the fact that teachers see the need to inform students of the possibilities offered by these mobile apps, and to train them to learn how to use these devices correctly. This is because a large number of teachers and coaches see a high value in the use of mobile apps and digital resources to complement their work but perceive themselves as having a very low competence in the implementation of these tools [103,104]. Therefore, the training of teachers, coaches or managers may be an issue to consider if the aim is for these tools to be used as a complement to the teaching and learning process of adolescents.

In response to the second question regarding the characteristics and aspects that should be present in a mobile app aimed at adolescents, it is necessary to design a gamified app that, in addition to recording physical activity, include information on other healthy habits such as eating and sleeping. Moreover, both adolescents and professionals highlighted the importance of including challenges, competitions, and being able to observe the progress made, a multimedia section, as well as information and healthy recommendations. Specifically, the adolescents requested aspects related to privacy and obtaining rewards, while teachers, managers and experts asked for the inclusion of feedback in the app, the facilitation of work through a simple and quick interface, and that they be trained on the possibilities of using these apps with adolescents.

The present research is not without limitations. The use of the focus group, although a valid method for collecting information based on semi-structured interviews and spontaneous participation, may lead to a less in-depth approach to the problems raised than other methodologies such as interviews. The selection of the secondary school for accessibility may also mean that the findings obtained in the adolescent focus groups have limitations when extrapolated to students from other schools who may have different casuistries; therefore, it would be interesting to analyze this topic in students from other contexts. And, the sample used, although large as compared to previous research, was still small and could not be generalized to all adolescents and professionals in the field, so future research is needed to answer the questions raised in this study.

The practical implications derived from the present research that can be considered by app developers, educators, and policymakers are a) gamification seems to be a fundamental element to increase motivation and engagement for physical activity apps for adolescents. However, its inclusion usually entails an increase in the requirements of the devices on which the app is used, so it is an aspect to consider; b) the inclusion of challenges, competitions and rewards is an element that does not entail such a drastic increase in the requirements of the app and that maintains motivation and interest in the app, so its inclusion in an app for adolescents is fundamental and necessary; c) the professionals who work with adolescents are a fundamental element to promote the use of the app, so they must be included in an app designed specifically for adolescents. However, this makes it necessary to consider that these professionals will only use the app if they consider it to be simple, in terms of interface, and if it facilitates their day-to-day work. Therefore, the inclusion of a specific section for teachers within the app should be considered; and d) gender issues, although they do not appear to be decisive, may influence whether the app is ultimately used by a greater or lesser number of users. Therefore, all those issues (logos, characters) that may lead to associate the app with a certain gender should be avoided.

With the data obtained, future research should try to propose longitudinal designs to analyze the adherence generated by this type of app over time according to the different elements included (challenges, competition, gamification). In addition, it would be possible to analyze in depth whether there really are gender differences in the use of certain physical activity apps and, specifically, in step-counting apps, since there is no scientific evidence to date to corroborate this. And, of course, an app should be designed that includes the contents mentioned in this manuscript and that is adapted as much as possible to adolescents. This prototype should be used to analyze whether the changes achieved in body composition, physical condition or psychological state are similar to those obtained with the apps already available or whether there are significant improvements in comparison.

## Conclusion

Based on the results obtained, it can be concluded that adolescents, teachers, coaches, managers, and experts consider that the apps currently available are more useful for use outside school but have limitations both in this setting and at school. These apps are not designed for adolescents, which considerably limits their use, as well as the possibilities of establishing healthy habits with them. There are discrepancies as to whether they are designed for males or females, but the fact that they are gamified seems to be one of the main aspects to take into consideration. In addition, the inclusion of information and healthy recommendations; challenges, competitions, and being able to observe the progress made; a multimedia section in which to find photos and videos; and the possibility of individualizing within the app is relevant for adolescents, teachers, coaches, managers, and experts. Furthermore, professionals asked for a quick and simple interface that facilitates and automates their work and that can be used offline so that it can be included during classes.
